# Women living with HIV/AIDS (WLHA), battling stigma, discrimination and denial and the role of support groups as a coping strategy: a review of literature

**DOI:** 10.1186/s12978-015-0032-9

**Published:** 2015-06-02

**Authors:** Vikas Paudel, Kedar P Baral

**Affiliations:** School of Health, Education and Community Studies, University of Northumbria, New Castle City, UK; Department of Community Health Sciences, Patan Academy of Health Sciences, Kathmandu, GPO Box 26500, Nepal; MIRA, YB Bhawan, Kathmandu, PO Box 921, Nepal

**Keywords:** HIV/AIDS, Women, Stigma, Discrimination, Coping strategy, Support group

## Abstract

**Background and objectives:**

Women living with HIV/AIDS, in particular, have been positioned as a latent source of infection, and have captivated culpability and blame leading to a highly stigmatised and discriminated life. Despite the situation, women and their particular concerns have largely been ignored in HIV/AIDS research literature.

This review aims to examine and analyze the feelings, experiences and perceptions of Women living with HIV/AIDS (WLHA) and will also access the role of support group as a coping strategy on the basis of 7 primary researches conducted in or on different parts of the world.

**Methodology:**

A systematic literature search was carried out on major data bases ASSIA, CINAHL, Science Direct, Web of Knowledge, Wiley Inter Science, AMED, Pub Med/Bio Med Central, MEDLINE and Cochrane Library. The articles included for review purpose were gauged against the pre-defined inclusion/exclusion criteria and quality assessment checklist resulting in a final 7 papers.

**Findings/results:**

The findings were compiled into five thematic areas: (1) Disclosure as a sensitive issue; (2) Stigma and Discrimination associated with HIV/AIDS and the multidimensional effects on women’s health and wellbeing; (3) Internalised Stigma; (4) Women living with HIV/AIDS experiences of being rejected, shunned and treated differently by physicians, family and close friends; (5) Support Group as among the best available interventions for stigma and discrimination.

**Conclusion:**

Support groups should be offered as a fundamental part of HIV/AIDS services and should be advocated as an effective and useful intervention. Further research is needed to examine the effect of support groups for women living with HIV/AIDS. A community based randomised controlled trial with support group as an intervention and a control group could provide further evidence of the value of support groups.

**Electronic supplementary material:**

The online version of this article (doi:10.1186/s12978-015-0032-9) contains supplementary material, which is available to authorized users.

## Background

### HIV and AIDS

AIDS- The Acquired Immune Deficiency Syndrome- is defined as a disease indicative of a defect in cell mediated immunity occurring in a person with no known cause for immune deficiency other than the presence of Human Immune Virus- HIV [1–3]. AIDS is the fourth and advanced stage of HIV infection and this condition gradually diminishes the effectiveness of the immune system and leaves individuals susceptible to opportunistic infections [4–6]. Identified in 1981, HIV/AIDS within a single generation has become the most sweeping and detrimental epidemic the world has ever experienced [7, 8].

### HIV/AIDS and women

HIV/AIDS is now recognised as a disease that affects women as well as men [9–11]. Women are increasingly at high risk of becoming HIV positive due to biological vulnerabilities, low socio-economic status, dominant sexual practice of males and epidemiological factors [12–14]. Men are more efficient at transmitting HIV to women than women are to men, and women are biologically more vulnerable to HIV infection than men [15, 16]. As the receptive partner, a woman has a larger mucosal surface exposed during sexual intercourse. In addition, semen contains a higher volume and concentration of virus than vaginal or cervical secretions. The risk of being infected with HIV during unprotected sex is two to four times greater for a woman than for a man [17]. Women often have little power or control over decisions relating to the sexual behaviour of their partners, such as condom use and safer sex, and over access to primary prevention information [18–20]. Women are also vulnerable to coerced sex including marital and non-marital rape, sexual abuse in and outside the family [21]. This sexual subordination of women makes it difficult for them to protect themselves from sexually transmitted infections (STIs), including HIV infection [12].

### Women: HIV/AIDS, stigma, discrimination and denial

Life for HIV infected women is never easy; they manifest profound physical and psychological consequences [9, 22]. Women bear a ‘triple jeopardy’ impact of HIV/AIDS: as person infected with HIV, as mothers of child, and as carers of partners, parents or orphans with AIDS [13]. Women living with HIV/AIDS (WLHA) are at particularly high risk of living a painful, shameful life of exclusion [23]. Millions have been rejected from their family, friends and partners, thousands have lost their lives and thousands have been unable to live their life [24–26]. In spite of the burden of disease the world is paying less attention to the issues raised by WLHA. Their voices remain unheard [27]. Since it was first identified, HIV/AIDS has been linked with ‘sexual misbehaviour’ and ‘promiscuity’ [28] contributing to the high level of stigma and discrimination associated with it. Women are often even more susceptible to the stigma associated with HIV/AIDS [10, 14, 27, 29] and are frequently referred to as ‘vectors’, ‘diseased’ and ‘prostitutes’. Discrimination for women can dispirit them from seeking vital medical and psychological care they need during the illness [30]. HIV stigma in women is associated with rejection from friends and family, society, feelings of uncertainty and loss, low self esteem, fear, anxiety, depression and even suicidal ideation [31–33]. Sandelowski *et al.* [34], in a meta-synthesis of 93 reports of research studies, examined the issue of stigma and discrimination in women living with HIV/AIDS. While only 16 of 93 reports reviewed were specifically focused on stigma, their study revealed that for women, living with HIV/AIDS meant living with panic, and the painful effects of stigmatisation and discrimination including social rejection, denial, even violence within family and community. The rejection and discrimination extends to treatment by health care professionals. The study also highlighted that women are facing higher levels of discrimination from society just because they are women.

### Coping strategy: support group

Coping with the multidimensional and complex effects of stigma and discrimination is never an easy task for women, particularly where their social status is already low. HIV-positive women have inimitable needs and concerns which can be best addressed with support groups [26]. Involvement in a support group has been correlated with reducing apprehension, depression, loneliness and isolation [35]. Support groups offer supportive environment for women with HIV to express their suppressed feelings in the company of women in the same situation. It also facilitates the sharing of strategies for securely disclosing HIV status, builds a network of friends to socialise with and provides emotional support [36, 37]. Gray [38] argued that women in support groups become empowered to value themselves and their life and make small steps to improve their life both emotionally and physically.

### Women and HIV/AIDS in Nepal

Though the known number of women with HIV is less than the number of known cases among men, it is escalating and the epidemic is hitting women hard in Nepal. Gender inequities and poverty have increased the vulnerability of women to HIV risk behaviors and exposure [39]. Women’s role in the community, low standing in the social hierarchy, low control in decision making, economic dependence, and low levels of literacy, have made them more prone to stigma and discrimination at the community and family level as well as by health personnel [39, 40]. Due to very limited published evidence to draw the scenario of women living with HIV/AIDS in Nepal, this systematic review will also critically examine the literature from other countries seeking to extrapolate similar conditions and circumstances of women in Nepalese context. The systematic review will also explore evidence to provide recommendations to improve the life of women living with HIV/AIDS in Nepal.

There have been many systematic reviews carried out on HIV/AIDS, but there are very few that have managed to expose the pains and suffering of women living with HIV/AIDS. Among that little puddle, Wojcicki [41] studied socio economic status as a risk factor for HIV infection in women and McCoy et al. [42] focused on behavior change interventions to prevent HIV infection in women. Still, reviews which examined the feelings, experiences, pains, success and challenges of women living with HIV/AIDS and role of support groups are rare. Moreover, the research in this area is predominantly quantitative research. Thus this review has stepped out in terms of using primary qualitative research to explore and examine the barriers and challenges, especially stigma, discrimination and denial faced by WLHA and also assess the role and impact of support groups as a coping strategy for WLHA.

## Methodology

### Search strategy

A systematic search was conducted using various online databases through the library search engine (NORA) at Northumbria University. NORA is devised to provide a wide range of all the available electronic information. Databases were searched with the help of key words. The data bases included ASSIA (Allied Social Sciences Index & Abstracts), CINAHL with full text (Cumulative Index to Nursing and Allied Health Literatures), Pro Quest Nursing Journals, Science Direct, Web of Knowledge, Wiley Inter Science, AMED (Allied and Complementary Medicine Database), Pub Med/Bio Med Central, MEDLINE and Cochrane Library. In addition, 14 studies were obtained through interlibrary loan due to the inability to access electronically. Similarly, the references and bibliographies of the 36 most relevant studies were also searched and produced 28 more articles, of which 20 were excluded immediately based on the exclusion criteria. The database searches finally resulted in 36 applicable papers with 8 additional papers identified through the review of the reference lists of those 36 articles and 1 selected through interlibrary loan. Therefore a total of 45 papers were identified as relevant to the topic area and obtained in full text for assessment of inclusion–exclusion in the review.

### Inclusion and exclusion criteria

Inclusion and exclusion of the articles were done on the basis of the following criteria:

- *Study design*: As the objective of the review was to explore the experiences, perceptions and feelings of women living with HIV/AIDS rather than ‘size of the issue’, only qualitative studies were included in the study. Qualitative studies employing any of its methods/approaches; focus group discussion (FGDs), key informant interviews, phenomenology, ethnography, case studies were included in the review.
- *Publication time scale:* To include recent and up to date articles for the review, only articles from 1995 onwards were included in the review.
- *Language:* Articles published in English language were considered for inclusion in this review.
- *Availability of full text:* Studies available in full text form from one or more following databases were included in the study. Databases include: ASSIA, CINAHL with full text, Pro Quest Nursing Journals, Science Direct, Web of Knowledge, Wiley Inter Science, AMED, Pub Med/Bio Med Central, and MEDLINE.
- *Research population/Type of participants:* Women Living with HIV/AIDS were taken as the research population for the review. Studies using suspected/undiagnosed women with HIV/AIDS were strictly excluded. There was no age limit for women with HIV/AIDS.
- *Location of research paper/article:* Articles whose abstracts were available from two or more of the selected databases (listed above) were included for the review.
- *Sample size:* Qualitative studies involving 8 or more women living with HIV/AIDS as the study population were included for this review.
- *Journal subset/type:* Peer reviewed articles were included in this review.

### Data extraction and quality assessment

A standard data extraction form was designed for this review to evaluate the seven selected articles individually. The relevant information from each study was extracted and recorded in the form by first author. All the extracted forms were checked by the last author to ensure precision, thoroughness, and consistency of the extraction procedure and records.

Detailed quality assessment was carried out individually for each 20 research article. All those 20 articles were assessed under 12 criteria/areas to filter them into final 7. Areas for quality assessment were;

- C1: Must meet previous all Inclusion and Exclusion Criteria.
- C2: Inclusion and Exclusion Criteria clearly stated in individual study.
- C3: Aims of the study.
- C4: Methodology of the study.
- C5: Justification for research.
- C6: Ethics.
- C7: Sampling methods.
- C8: Consent of the participants.
- C9: Bias.
- C10: Drop out/Refusal of/from participants.
- C11: Data Analysis.
- C12: Outcomes/results.

Each of the criteria was marked 1 and for the inclusion in the final 7 review list, the article should score 10 or more than 10. Included and excluded articles based on quality assessment checklist are provided in Additional file 1.

## Results

### Search results

After application of quality assessment checklist for 20 potential studies, seven studies were finally selected for the review. Those seven are as follows:

- S1: Metcalfe et al. [43] Meeting the needs of women living with HIV.
- S2: Thomas et al. [44] Impact of HIV/AIDS on Mothers in Southern India: A qualitative study
- S3: Medeley et al. [45] Disclosure Outcomes, Coping Strategies and Life Changes among Women Living with HIV in Uganda.
- S4: Lawless et al. [10] Dirty, Diseased and Undeserving: The positioning of HIV positive women
- S5: Balaile et al. [46] Poverty and devastation of Intimate Relations: Tanzanian women’s experience of Living with HIV/AIDS.
- S6: Carr et al. [47] Stigma: A health barrier for Women with HIV/AIDS.
- S7: Liamputttong et al. [48] HIV and AIDS, stigma and AIDS support groups: Perspectives from women living with HIV/AIDS in Central Thailand.

The study identification flowchart (Additional file 2) demonstrates the process of identifying the above final papers for this review.

### A narrative synthesis of included studies

The studies which have been included in this review were diverse. They were different in many ways methodologically and also in terms of results. One way of doing a review would entail focusing on the most methodologically sound studies, but this can only work if there is a large enough source of homogeneous and robust studies to draw upon [49]. In this instance as anticipated, only a relatively small number of studies were found. These were of varying quality. A narrative synthesis was therefore been undertaken.

### General characteristics and study designs

Selected studies were published between 1998 and 2009 carried out in various countries representing different parts of the world. S1 was carried out in Canada, S2 in India, S3 in Uganda, S4 in Australia, S5 in Tanzania, S6 in United States of America (USA) and S7 in Thailand. Despite some differences in words used, overall, the aim of each study was to explore the perceptions and experiences of women living with HIV/AIDS, the difficulties/challenges in their life and the importance of support groups.

As the aim of all the seven selected studies was to explore the perceptions and experiences of women living with HIV/AIDS, they employed a variety of qualitative methodologies. S5, S7 and S1 used phenomenological-hermeneutic design, a qualitative methodology which is used to grasp the meaning of lived experience by interpreting narrative interviews transcribed as a text [50]. S6 employed ethnographic methodology, a qualitative design in which the researcher describes and interprets shared and learned patterns of values, behaviours, beliefs, perceptions and experiences of a culture sharing group. Studies S1, S3. S4, S5, S6, S7 used in-depth interviews while S2 used FGDs as data collection method. Each has their own strengths as a qualitative method.

### Participants, sampling and methodology

WLHA were the participants in each study. In total, 141 women living with HIV/AIDS were involved in the studies. The number in each study varies with S1 having the smallest number of participants 8 and S2 having greatest number 60. S3, S4, S5, and S6 involved 9,10,30,24 participants respectively. The participants in each study differed in terms of marital status, education level and number of children. All study participants were 18 and above. Age of the participants in S2, S3, S4, S5, S6, and S7 was 23–42, 18–49, 22–55, 28–45, 27–56, and 20–45 respectively. However age category of the respondents is not mentioned in S1. Majority of the women in the study had educational qualification up to primary level.

Method of recruitment varied between the studies. In, S1 information letter were distributed, S5 recruited participants face to face in three different settings, S6 recruited using volunteer and networking sampling techniques, S4 through distribution of flyers, S3 recruited from 10 antenatal clinics, S2 from maternity hospital and S7, through advertising on bulletin boards at hospitals and contacts made by Thai co-researcher.

Consistent criteria for inclusion were more than 18 years of old, and diagnosed HIV positive. The term women living with HIV/AIDS (WLHA) in all studies have been used to describe only diagnosed or documented cases rather than suspected or undiagnosed. S2 and S5 used ability to read and write in the local language as an inclusion criteria. S5 identified that women should be diagnosed with HIV for at least 1 month, however S5 had the similar criteria for minimum 1 year. S2 used no evidence of cognitive dysfunction as assessed by investigator as an inclusion criterion. Similarly, S3 used women that were currently pregnant or had given birth to the baby within the previous year as one of the inclusion criteria. Sampling procedures in 6 studies was purposive random sampling. However S1 used convenience sampling.

### Data collection and analysis

Data collection in all the studies were conducted in private settings and inform consent was obtained in every study prior to the data collection. All of the interviews and focus group discussions were tape recorded with the consent of the participants. Interviews in average for all studies lasted from 45 minutes to 1 hour. Each FGD lasted between 60–75 minutes. Data analysis in all studies was different. In S1 and S7 recorded data were transcribed and developed into themes. In S2 NVIVO was used as a tool to store, organise and analyse the data from FGDs. S3 used iterative coding process to analyse the data. Similarly, S4 used principles of grounded theory to analyse the transcripts of interviews. In S5 the data were analysed phase by phase using phenomenological-hermeneutic method. While data analysis process in S6 was concurrent with data collection.

### Analysis on thematic areas

The selected primary papers were examined for five themes listed below. The key themes of six studies are all interlinked, due to the common factors which characterise the population of concern (WLHA). The following cross-study themes were categorised:

1. *Disclosure-a sensitive issue:* Included studies found out that the secrecy/fear of disclosure was killing women with HIV/AIDS faster than the disease. S3 revealed that despite numerous benefits to HIV disclosure such as increased social support and kindness and easy access to health care treatment, there are also numbers of potential risks, especially for women. These include abandonment and relationship termination, stigma and discrimination and emotional abuse. And this had kept women in dilemma whether to disclose their status or not. In S2 the majority (95 %) of participants agreed that disclosure was a sensitive issue. Fear surfaced, such as reaction of in-laws and the impact of disclosure on their children. Fear of stigma and other negative outcomes has been cited as in S1, S3 as one of the barrier to HIV disclosure among women. S6 revealed that not disclosing the status is associated with more deterioration of their health status. Firstly women hesitate and fear to receive the care and treatment needed to them. Secondly they themselves become the victim of stress, depression, inferiority feeling, guilt, and suicidal feelings. Similarly, S7 revealed that women were living in isolation and were utilizing limited health services due to the fear of disclosure. S4 found out that women also fear of disclosure because they didn’t want to jeopardise the lives of children.
2. *Stigma and discrimination: multidimensional effects on women’s health and wellbeing:* S6 revealed that women living with HIV/AIDS countenance physical, social, emotional and spiritual difficulties in dealing with discrimination and denial from family, friends, community and health professionals. Discrimination and denial range from refusal by their partners, physical violence, rejection from family and society, loss of employment, financial difficulties and many more. S7 concluded that stigma and discrimination brought psychological problems like low social esteem, anxiety disorder, suicidal ideas and emotional insecurity. S2 found that stigma and discrimination has restricted them from receiving health care facilities worsening their health condition. S5 presented the experiences of women who were facing financial difficulties and were struggling hard to maintain their life due to the loss of job. S4 revealed that infection with HIV/AIDS in women brought loss/end of social life and they were living an isolated and miserable life. Similarly, S4 found that stigma and discrimination brought devastating mental, social, spiritual and economic consequences in women living with HIV/AIDS.
3. *Internalised stigma*: Self stigma was hitting women more than stigma and discrimination from friends and society. S2 revealed that self stigma lead women to feeling of self blame, guilt which may lead to isolation, hesitation to seek health services, ultimately deterioration of their health status. It also led to fear of disclosure of HIV/AIDS which led to stress, depression, inferiority feeling, anxiety disorder, feeling of guilt, and sometime the suicidal attempt. S4 and S1 described the experiences of women in which she felt she was contaminated and polluted and not able to face the community to fight against HIV/AIDS.
4. *Women living with HIV/AIDS were rejected, shunned and treated differently by physicians, family and close friends:* All the studies described about the differences in taking and treating women after the disclosure. S4 concluded that infection with HIV appears to imply failure in the expected traditional role for women as carers and moral guidance and are either damned whores or God’s police. S7 revealed that for women living with HIV/AIDS meant living with panic, and hurtful effects of stigma and discrimination including social rejection, denial even violence in family, community and health care professionals. The same presented the experiences of some women living with HIV/AIDS who had to lose the job, were rejected from the family and some faced violence from the family. S1 argued that women are facing extra discrimination from the society or from elsewhere just because they are women. S6 concluded that women with HIV/AIDS are living a painful, shameful and excluded life. S3 revealed that discrimination and denial range from refusal by their partners, physical violence, rejection, and segregation from family and society, exclusion from housing, loss of employment, financial difficulties and psychological problems.
5. *Support group act as among the best interventions for stigma and discrimination:* All studies highlighted the importance of support groups to deal with the multidimensional effects of stigma and discrimination. S7 advocated the role of support groups which provide opportunities to rehearse disclosure and where women become empowered to value themselves. S1, S2 also stated the importance of support group as a coping strategy. These studies pointed out that support groups are mainly associated with decreasing isolation and feeling of shame and increase network of friends to mingle with. S3 pointed out that support group can play an important role in it bringing wide range of women in a common place with a commons situation. S4 advocated support groups as the most important psychological intervention for women living with HIV/AIDS and pointed out that support groups provide anticipatory guidance which potentially decreases the fear.
  The concept of support group can be well applicable to the Nepalese context but there are limited numbers of support groups in Nepal working to improve the life of women living with HIV/AIDS especially in remote and rural areas. Overall access to all WLHA to the functional groups round the year is far less than adequate.

## Discussion

This systematic review of the literatures observed that women living with HIV/AIDS in a number of different countries are experiencing high levels of stigma and discrimination from friends, family, community and even health workers. Support groups were found to decrease isolation and feelings of shame, increase the network of friends to mingle with, create mutually empathetic relationships, improve self-care behaviours, and decrease risk behaviour for re-exposure to HIV.

One of the biggest concerns raised from the review is the discrimination faced by WLHA from health workers. The findings from this review are congruent with some of the previous research [51, 52] on discrimination by health workers. Health workers are often community role models for conveying any health related concepts and ideas. Their attitudes and behaviours can shape the way members of the community treat persons suffering from health problems by challenging stereotypes and discriminatory attitudes. Health care workers must be educated about their responsibility so they do not perpetuate negative attitudes towards persons living with HIV.

Disclosure is another important issue with women living with HIV/AIDS. It has been an obstacle for WLHA as not disclosing might deprive them of the opportunity for getting proper attention, care and access to facilities from family and health institutions. But in the mean time, it might become a burden when those persons do not get WLHA right. North and Rothenberg [53], in their work, examine domestic violence against women living with HIV discussed the experience of two women who were shot as result of disclosing that they were HIV-positive.

Understanding the multifaceted stigma and discrimination requires the understanding of all factors associated with HIV stigma in HIV-positive women. Wagner et al. [54] identified education as one of the major factors that increases stigma among women. Leasure *et al.* [55] identified employment level/financial situation and educational level as major perpetuators for discrimination against WLHA. Family Health International-Nepal [39] identified women’s role in the community, low standing in social hierarchy, and low control in decision making, economic dependence, and illiteracy as major perpetuators for stigma and discrimination towards women with HIV/AIDS. Chung & Margraw [56] discussed that women’s experience of HIV infection is inseparably concurrent to their experience as women. The study argued that woman’s social roles, especially roles involving reproduction and childbearing, are a triggering factor for HIV infection in women and also stigma and discrimination. Issiska et al. [57] found that sharing sero-status with their partner was highly related to the educational level of the HIV infected women. This means empowering women with education and income generating works may help in boosting their morale and confidence which ultimately helps in fighting stigma and discrimination.

The findings of this review on support groups are harmonious with a previous review [58]. However, the review also debates on the process, content, outcomes of support groups, structure of the group, number of participants, and involvement of men in the process of the group formation and function should be clearly thought about to maintain social support mechanism and sustainability. The review also recommends that groups and interventions should be designed properly to meet the need of all the diverse range of people coming in the group who might vary in needs and interest.

### Implication of finding for public health practice and research with reference to Nepalese context

The findings from this review can be highly relevant in the context of Nepalese women. Despite demographic, cultural and geographical variation between included studies and the Nepalese context, findings fairly reflect the challenges faced by Nepalese women living with HIV/AIDS. Most of the findings can also be applied to some South Asian countries such as India, Bangladesh, Pakistan, and Sri lanka where the condition of WLHA is quite similar to Nepalese women.

Women-oriented programs must raise awareness and understanding of the plight of women in Nepal [39]. Awareness of various facets and dynamics of HIV/AIDS can be a powerful tool to reduce the impact of the misconceptions or myths regarding HIV/AIDS in women [59]. On the other hand, it can build women’s level of knowledge, means of caring for themselves, ways of tackling their health problems (such as opportunistic infection) and of course builds their self esteem, confidence, and power to fight back against stigma and the virus itself [60]. Enrolment campaign for girls should be the main focus in developing countries such as Nepal as they can build their base of knowledge and confidence on various dynamics of HIV/AIDS from their childhood. Likewise, support services including economic and income-generation skills development, educational programs, transitional economic and housing support, counseling and referral services, and consideration of support for women with children and for orphaned children should be targeted in programs for WLHA [39]. In addition, structural interventions like anti-discriminatory laws, treating HIV/AIDS as a medical condition, and easy availability of ARV are required to change the social environment and climate in which people live with HIV/AIDS. Similarly, health care personnel at all levels need to be equipped with intensive training to sensitize them on need to treat WLHA without discrimination and provide them quality of care [61]. Finally, support groups for women living with HIV/AIDS should be offered as a fundamental part of HIV services and should be advocated as an effective and useful intervention. Likewise, the Government of Nepal should provide financial and technical assistant for establishing and sustaining support groups and policy should incorporate these issues.

### Limitations of the review

This review attempted to follow the transparent and systematic approach to identify methodologically sound papers for being appraised. However, the number of included studies is small and rigorously-designed studies on the given topic seem to be rare. By virtue of pre-defined search criteria and selected databases for literature searches, the chances of missing some valuable studies not indexed in those databases and published in non-English languages cannot be ignored.

### Recommendations

1. Promoting and protecting women’s human rights: Still in many parts of the world and in Nepal there prevail major inequalities between men and women in various aspects of life ranging from education, access to health services, employment, power within the relationships and many other basic human rights. Prior to end stigma and discrimination it’s important to secure their basic human rights.
2. There needs to be support services especially for female PLWHA and their children.
  These services should include: economic and income-generation skill development, educational programs, transitional economic and housing support, counselling and referral services, and consideration of support for women with children and for orphaned children.
3. Health care personnel at all level need to be equipped with intensive trainings to sensitize them on need to treat WLHA without discrimination and provide them quality of care.
4. Structural interventions like anti-discriminatory laws, treating HIV/AIDS as a medical condition, including easy availability of ARVs are required to change the social environment and climate of AIDS and ultimately to lessen the stigma and discrimination associated with WLHA.
5. Creating women’s participation in building their own health: Women should be encouraged to participate in their own health. Women should be motivated to talk and discuss their status openly in an attempt to de-stigmatise the disease and provide support and care for those living with HIV/AIDS. It can increase their knowledge on the particular health problem, teach to be self dependent on their health, and make control over their decision.
6. Support groups for women living with HIV/AIDS should be offered as a fundamental part of HIV services and should be advocated as an effective and useful intervention. Government should provide financial and technical assistant for establishing and sustaining support groups and policy should incorporate these issues.

### Implication for future research and practice

Well-designed research is needed in both high income and low income countries to examine the effect of support groups for women living with HIV/AIDS. A community based randomised controlled trial with support group as an intervention and a control can be applied to see the effect. Research works should also focus on impacts of gender on women’s experiences with HIV/AIDS in Nepal. The research should raise the issues of power within a relationship, societal role of women, educational level, control in decision making, and social hierarchy which are critical factors for stigma and discrimination for women living with HIV/AIDS [27, 62]. Further research should be conducted on families with members who are living with HIV, the effects of the illness on their relationships as well as planning for future needs. Nutritional status of WLHA and their children could be another area of exploration.

## Conclusion

Challenging stigma, discrimination and denial in the context of HIV/AIDS requires commitment at all levels including governments, civil society, communities and individuals. HIV/AIDS is much more than a health problem. It touches human conditions, human security, human rights and social and economic development. A human rights framework is essential to encourage a reduction in HIV/AIDS-related stigma and discrimination. As an essential human rights issue, gender equality can be at the forefront of development and security as well as building healthy populations. The spread and impact of HIV is fuelled when human rights are violated. Nowhere is this more evident than with respect to the inequality evident among Women Living with HIV/AIDS. Hence, respect and fulfillment of human rights is critical to lessening the adverse impact of the disease.

## Additional files

Additional file 1:
**Quality assessment of the 20 studies with potentiality to be included in the review.**
Additional file 2:
**Study Identification Flow Chart.** Potentially relevant citations identified through initially comprehensive electronic search (N= 769 citations with title and abstracts).
