# Supplementary material for: Women living with HIV/AIDS (WLHA), battling stigma, discrimination and denial and the role of support groups as a coping strategy: a review of literature
Source: Reprod Health. 2015 Jun 2;12:53. doi: 10.1186/s12978-015-0032-9 (PMC4467680; doi:10.1186/s12978-015-0032-9)
Supplement: Additional file 1: — Quality assessment of the 20 studies with potentiality to be included in the review. [file 12978_2015_32_MOESM1_ESM.doc]

| **Studies** | C1* | C2* | C3* | C4* | C5* | C6 | C7* | C8* | C9* | C10 | C11* | C12* | Total |
| --- | --- | --- | --- | --- | --- | --- | --- | --- | --- | --- | --- | --- | --- |
| **Included** | | | | | | | | | | | | | |
| Lawless et al 1996 | √ | √ | √ | √ | √ | √ | √ | √ | √ |  | √ | √ | 11/12 |
| Medley et al 2009 | √ | √ | √ | √ | √ |  | √ | √ | √ | √ | √ | √ | 11/12 |
| Liamputtong et al 2009 | √ | √ | √ | √ | √ | √ | √ | √ | √ |  | √ | √ | 11/12 |
| Thomas et al 2009 | √ | √ | √ | √ | √ | √ | √ | √ | √ |  | √ | √ | 11/12 |
| Carr and gramling 2004 | √ | √ | √ | √ | √ |  | √ | √ | √ |  | √ | √ | 10/12 |
| Metcalfe et al 1998 | √ | √ | √ | √ | √ |  | √ | √ | √ |  | √ | √ | 10/12 |
| Balaile et al 2007 | √ | √ | √ | √ | √ |  | √ | √ | √ |  | √ | √ | 10/12 |
| **Excluded** | | | | | | | | | | | | | |
| Ross et al 2007 | √ |  | √ | √ |  |  | √ | √ | √ |  | √ | √ | 8/12 |
| Dane, B. (2002) | √ | √ | √ | √ | √ |  |  | √ | √ |  | √ | √ | 9/12 |
| Marenco, M.O., Samost, L (1999) | √ |  | √ | √ | √ |  |  |  | √ | √ | √ | √ | 8/12 |
| Zhou, Y.R. (2010) | √ | √ | √ | √ | √ |  | √ |  |  |  |  | √ | 7/12 |
| Hodge, D.R and Rohy, J.L (2010) |  | √ | √ | √ |  |  |  | √ |  |  | √ | √ | 6/12 |
| McCall et al. (2009) | √ |  | √ | √ |  |  | √ | √ |  |  | √ |  | 6/12 |
| Bond et al (2002) | √ | √ | √ | √ | √ |  | √ | √ |  |  |  | √ | 8/12 |
| Bennett, J (2007) | √ |  | √ | √ | √ |  | √ | √ |  |  | √ |  | 7/12 |
| DeMarco et al (1998) | √ | √ | √ | √ | √ |  | √ |  |  |  | √ | √ | 8/12 |
| Mayers et al (2004) | √ | √ | √ | √ | √ |  |  | √ | √ |  |  | √ | 8/12 |
| Pittiglio, L and Hough, E (2009) | √ |  | √ | √ | √ |  | √ | √ | √ |  |  | √ | 8/12 |
| Leasure et al (2009) | √ | √ | √ | √ |  |  | √ | √ | √ |  | √ |  | 8/12 |
| Owens, S (2003) | √ |  | √ | √ | √ |  | √ | √ |  |  | √ | √ | 8/12 |

**Appendix 1: Quality assessment of the 20 studies with potentiality to be included in the review**
