# Supplementary material for: Women living with HIV/AIDS (WLHA), battling stigma, discrimination and denial and the role of support groups as a coping strategy: a review of literature
Source: Reprod Health. 2015 Jun 2;12:53. doi: 10.1186/s12978-015-0032-9 (PMC4467680; doi:10.1186/s12978-015-0032-9)
Supplement: Additional file 2: — Study Identification Flow Chart. Potentially relevant citations identified through initially comprehensive electronic search (N= 769 citations with title and abstracts). [file 12978_2015_32_MOESM2_ESM.doc]

**Appendix 2**

**Study Identification Flow Chart**

Potentially relevant citations Identified through initially comprehensive electronic search (N= 769 citations with title and abstracts)

Liberally scan title and abstract according to inclusion and exclusion criteria, **733** unrelated or irrelevant citations

●References and bibliographies of the **36** most relevant studies produced **8** more relevant articles.

●**7** obtained from interlibrary loan, but just 1 relevant

**Retrieval of hard copies of potentially relevant citations (N= 45)**

●Further consider full text articles according to inclusion and exclusion criteria.

●**25** unrelated or irrelevant studies

**Potential Papers (N= 20 papers)**

Application of Quality Assessment Checklist, **13** unmet the quality assessment checklist

**Included Papers for Systematic review (N=7)**
